# Surgical treatment of recurrent urachal carcinoma with liver metastasis: a case report and literature review

**DOI:** 10.1186/s12957-016-1057-4

**Published:** 2016-11-28

**Authors:** Lukasz Paschke, Miroslaw Juszczak, Maciej Slupski

**Affiliations:** 1 Department of Liver and General Surgery, Nicolaus Copernicus University, Curie-Sklodowskiej 9, Bydgoszcz, 85-094 Poland; 2 Department of Urology, Nicolaus Copernicus University, Bydgoszcz, Poland

**Keywords:** Urachal carcinoma, Salvage surgery, Adjuvant chemotherapy

## Abstract

**Background:**

Urachal carcinoma is a rare malignancy with poor prognosis due to late presentation of the disease and its aggressiveness. Surgery remains the mainstay of therapy even in cases of disease recurrence. To the best of our knowledge, this is the first report of salvage surgery in the case of urachal carcinoma with liver metastasis.

**Case presentation:**

The patient was a young woman who suffered from locally advanced urachal carcinoma treated with en-bloc cystectomy, hysterectomy with bilateral adnexectomy, partial resection of the sigmoid colon, and partial resection of the rectus abdominis muscle with the fascia, skin, and umbilicus. Adjuvant chemotherapy with paclitaxel and carboplatin was applied. Two years after the treatment, she was diagnosed with a single liver metastasis and a local pelvic recurrence. In a two-step operation, the patient underwent right hemihepatectomy as well as resection of pelvic recurrence site and adjuvant chemotherapy with gemcitabine. Due to the disease progression, a complete resection of the lesions was not achieved and the response to chemotherapy was poor. The patient died of the disease after a year.

**Conclusions:**

Surgery is the first line of treatment for urachal carcinoma and should be always considered as an option in cases of disease recurrence. Radical initial surgical management, close patient surveillance, and prompt treatment of disease relapse may all contribute to prolonging patient’s survival.

## Background

The urachus is a structure connecting the allantois with a precursor of the urinary bladder during early embryonic development. It involutes before birth into a fibrous cord extending upward from the anterior dome of the bladder toward the umbilicus and forming a median umbilical ligament. It is a three-layered tubular structure lying in the Retzius’ space and varying in size from 3 to 10 cm in length and from 8 to 10 mm in diameter [1, 2]. The remnants of urachal tissue in the ligament may be a source of tumor growth. It is a rare type of malignancy with incidence below 0.5% of all bladder cancers and the annual incidence estimated to be 1 in 5 million people in general population [3, 4]. To date, no specific risk factors for the development of urachal carcinoma have been established. In the case of urachal mass of unknown character, the risk factors of its malignant character are age older than 55 years and the presence of hematuria [5].

The most commonly employed diagnostic criteria for urachal carcinoma are those proposed by Sheldon et al. These include (1) tumor in the dome of the bladder, (2) absence of cystitis cystica and cystitis glandularis, (3) predominant invasion of the muscularis or deeper tissues with a sharp demarcation between the tumor and surface bladder urothelium which is free of glandular or polypoid proliferation, (4) presence of urachal remnants within the tumor, (5) extension of tumor into the bladder wall involving the space of Retzius, anterior abdominal wall, or umbilicus, and (6) no evidence of a primary neoplasm elsewhere [4]. Simplified MD Anderson Cancer Center criteria published by Siefker-Radtke et al. include (1) location in the bladder dome or elsewhere in the midline of the bladder and (2) sharp demarcation between tumor and normal surface epithelium. Supportive criteria include enteric-type histology, absence of urothelial dysplasia, cystitis cystica or cystitis glandularis transitioning to the tumor, and absence of primary adenocarcinoma of another organ [6].

The majority of authors follow a staging system for urachal carcinoma proposed by Sheldon. Recently, a simplified system developed in Mayo Clinic has been proven to be of equal value. Table 1 compares both systems [4, 5].

**Table 1:** Comparison of urachal carcinoma staging systems

Stage	Sheldon	Mayo clinic
I	Confined to the urachal mucosa	Confined to the urachus and/or the bladder
II	Invasion confined to the urachus itself	Extension beyond the muscular layer of the urachus and/or the bladder
III		Metastases to the regional lymph nodes
IIIA	Extension to the bladder	
IIIB	Extension to the abdominal wall	
IIIC	Extension to the peritoneum	
IIID	Extension to the viscera other than the bladder	
IV		Metastases to non-regional lymph nodes or other distant sites
IVA	Metastases to the lymph nodes	
IVB	Metastases to distant sites	

Following the diagnosis of urachal carcinoma, the first line treatment consists of partial or radical cystectomy together with wide surgical excision of remaining urachal ligament, surrounding soft tissue, and umbilicus. Bilateral pelvic lymphadenectomy is optional [5–8]. As the cancer is usually diagnosed at an advanced stage, the incidence of local recurrences or distant metastases following the treatment is high. Due to the rarity of the disease, no clinical trials are available to assist in the choice of further treatment. Several chemotherapy regimens have been tested so far with limited success. Salvage surgical treatment with adjuvant chemotherapy in cases of disease relapse has been described in some studies as potentially prolonging patients’ survival [5, 9, 10].

## Case presentation

**Fig. 1:**
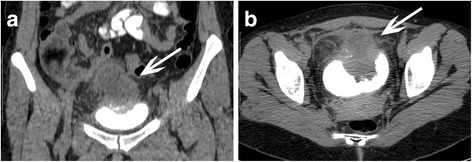
Pelvic CT scan at the time of diagnosis. Tumor mass is demarcated from the contrast-filled bladder (*white arrow*). **a** Coronal plane. **b** Transverse plane

A 34-year-old woman with a history of urachal carcinoma was referred to our department (Department of Liver and General Surgery) in January 2015 in order to surgically treat a disease relapse presenting with a liver metastasis. The patient was an otherwise healthy woman. The first symptoms of the disease occurred in December 2011 and included urinary frequency, dysuria, and episodic gross hematuria. In January 2012, the patient sought medical attention. The primary care physician diagnosed urinary tract infection and ordered antibiotics. After two months, an abdominal ultrasound was performed, however with an empty bladder, and the disease focus was not recognized. It was not until April 2012 that the patient came directly to the emergency department and was for the first time consulted by a urologist. At that point, the diagnosis of urinary bladder tumor was made. A CT scan showed a pathologic mass in the region of the bladder dome extending toward the umbilicus (Fig. 1) which is a typical appearance of urachal carcinoma [11]. It was adherent to the sigmoid colon, and there were signs of the surrounding adipose tissue and peritoneum involvement. No sites of distant metastases on abdominal CT scan and chest X-ray were noted.

In June 2012, the patient underwent radical cystectomy including hysterectomy, bilateral adnexectomy, partial resection of the sigmoid colon, and partial resection of the rectus abdominis muscle with the fascia, skin, and umbilicus. A urinary diversion with a Studer-type orthotopic ileal neobladder was constructed. Intraoperative histologic examination of two ileal lymph nodes was negative. However, a post-operative examination of these lymph nodes revealed one metastatic focus. The full specimen was described in pathology report as a 22 × 19 × 17 cm in size, with tumor extending between the uterus and umbilicus involving the urinary bladder and extending beyond its wall. On microscopic examination, a mucous-producing adenocarcinoma consistent with urachal carcinoma was diagnosed. The involvement of the sigmoid colon was caused by an inflammatory response. Other resected organs as well as surgical margins were free of neoplastic infiltration. The patient received four cycles of adjuvant chemotherapy with paclitaxel and carboplatin.

**Fig. 2:**
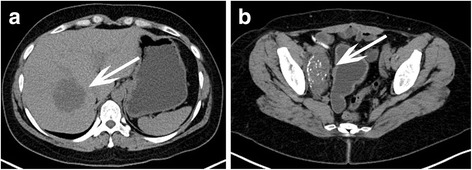
Abdominal and pelvic CT scan at the time of disease recurrence. **a** Liver metastatic focus (*white arrow*). **b** Local recurrence in the region of the right iliac vessels (*white arrow*)

**Fig. 3:**
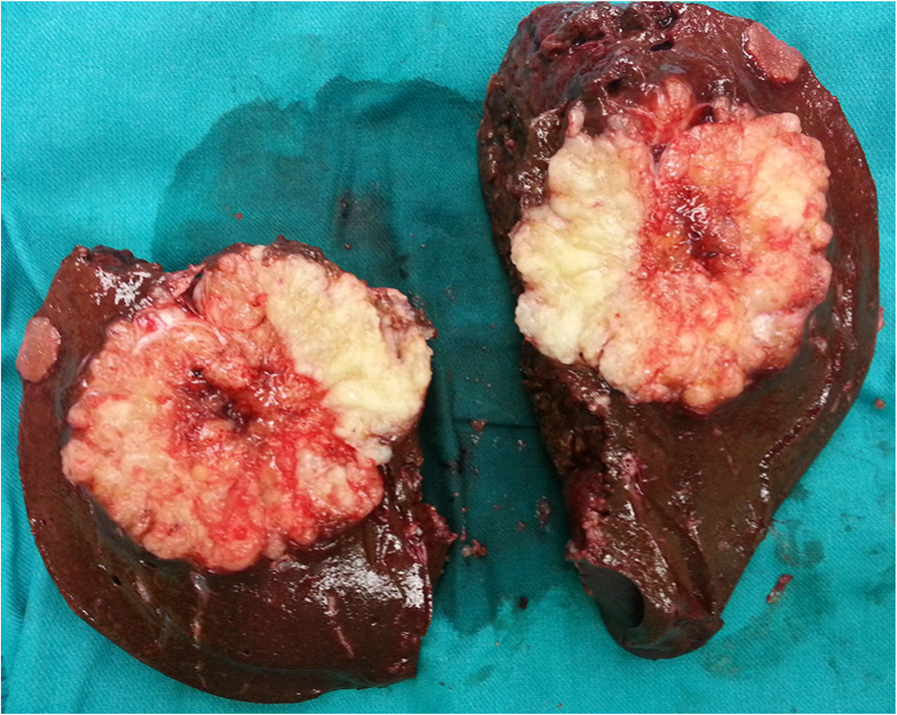
The right liver lobe with a metastatic tumor and a satellite focus

A follow-up CT scan performed a year after surgery (July 2013) showed no signs of disease recurrence. In November 2014, another CT scan revealed a hypodense mass in the right liver lobe of max 40 mm in diameter. A collection of fluid in the region of the right iliac vessels with calcifications was also noted (in a region where in previous imaging studies a simple lymphocele was described). A subsequent PET-CT scan of entire body proved a high probability of disease recurrence in the liver and in the region of the right iliac vessels (Fig. 2). A biopsy of the liver mass confirmed a focus of metastatic disease. After presentation of possible therapeutic options to the patient, she chose a surgical treatment. The surgery took place in January 2015. Intraoperative findings with the use of ultrasonography included a tumor of max 80 mm in diameter in the right liver lobe with a small satellite focus on the liver phrenic surface. Despite the probability of local recurrence in the pelvis, a right hemihepatectomy was performed. This decision was based on a large tumor size and a possible expansion into the vena cava inferior and liver hilum (Fig. 3). The pathology report revealed a metastatic urachal carcinoma and a positive surgical margin. Magnetic resonance imaging performed a month after the surgery confirmed enlargement of the pathologic mass in the pelvis. No other sites of the disease were noted. Patient was qualified to a second-stage surgical treatment and underwent an excision of the tumor mass in the region of the right iliac vessels in April 2015; however, complete resection has not been achieved.

Following the surgery, the patient received three cycles of adjuvant gemcitabine-based chemotherapy. Subsequent imaging studies showed gradual disease progression. The patient died of the disease in March 2016.

## Discussion

The presented case illustrates difficulties associated with the diagnosis and treatment of the urachal carcinoma. It took over 6 months since the patient started experiencing symptoms and sought medical attention until the treatment was instituted. This significant delay may have contributed to the advanced stage of the disease found at the time of the cystectomy. Literature review shows that the first urachal carcinoma symptoms include most commonly hematuria, followed by palpable mass in the lower abdomen, abnormalities in urine sample examination, and pain [12, 13]. Unfortunately, these occur mostly late in the course of the disease which leads to local cancer invasion or metastatic foci often being present at diagnosis [4]. In the study by Gopalan et al. among all 24 patients examined, there were no cases of Sheldon stage I or II tumors [14]. In another population-based study among 40 patients diagnosed with urachal tumor, only in one case cancer was confined to the urachus [15].

The pathologic study of urachal carcinoma in the majority of cases reveals an intestinal type adenocarcinoma with morphological variants including mucinous, enteric, signet ring cell type, and not otherwise specified (NOS) [16]. In the case of our patient, a mucinous type adenocarcinoma was found. This type, together with NOS adenocarcinoma, is the most frequent variant encountered [3, 4, 16, 17]. None of these variants, however, was found to have prognostic value, possibly due to paucity of patients [3, 14, 16]. In a recent study by Bisosonnette et al., the authors used a micro-RNA expression profiling in order to genetically differentiate between morphologic types of urachal carcinoma. No significant differences were found suggesting that urachal adenocarcinoma can be viewed as a single biological entity [18].

Due to the rarity of urachal carcinoma, no evidence-based standards of treatment have been developed. Wide surgical excision is widely regarded as the treatment of choice. A study by Henly et al. found no significant differences in the survival rate after radical cystectomy vs. partial cystectomy [19]. To date, published reports have not confirmed clear advantages of radical cystectomy (when partial cystectomy with safe surgical margins is technically feasible). However, the study by Gopalan et al. indicates a lower local recurrence rate in cases without bladder-sparing surgery [14]. In the last decade, an increasing amount of urachal carcinoma surgical treatment is performed using minimally invasive laparoscopic or robot-assisted technique. Although no studies have directly compared open surgery with minimally invasive techniques, the available case reports show that laparoscopic approach allows for radical surgical excision with the added benefit of reduced blood loss, decreased postoperative patient discomfort, and shorter hospital stay [20–22].

Another issue not standardized due to a low number of urachal carcinoma cases is the need to perform pelvic lymph node dissection (PLND). In the case of our patient, PLND was not performed. Intraoperative assessment of two lymph nodes brought a false negative result. To date, evidence is lacking to support performing PLND in all cases of urachal carcinoma. On the other hand, available data indicate that local recurrence of urachal carcinoma (including pelvic lymph nodes involvement) is the most frequent presentation of disease relapse [5, 14]. Moreover, the pelvic lymph nodes, together with the lungs, are the most frequent site of urachal carcinoma metastases [12, 14]. Based on available literature, performing PLND is described as optional. Some authors advocate sampling the lymph nodes, while others performing the extended PLND in all cases [5, 12, 13]. In the majority of recent descriptions of urachal carcinoma treatment, the PLND was performed similarly to the case of locally advanced bladder cancer where it is proved to benefit patients’ survival [13, 21, 23–25]. In the presented case, PLND could be of therapeutic value.

After receiving initial surgical treatment and adjuvant chemotherapy, the patient follow-up scheme included only abdominal CT scan a year after the treatment. The next abdominal CT scan, which raised the suspicion of the disease recurrence, was performed over a year later and, in fact, due to the patient’s symptoms—leg pain. There are no guidelines on follow-up scheme after radical cystectomy due to urachal carcinoma. Standards are adapted from treatment of muscle-invasive urothelial bladder cancer or aggressive adenocarcinomas. However, even in cases of these more prevalent diseases, no universally accepted standards supported by scientific cost-benefit analysis exist. Nevertheless, recent studies regarding follow-up schemes after radical cystectomy acknowledge the need for more frequent and multi-modal surveillance in cases of stage III and IV disease [26]. In cases such as the one described above, it would seem prudent to schedule abdominal CT scans more often and include different modalities such as abdominal ultrasonography and chest X-ray into the scheme.

The chemotherapeutic regimens received by the patient consisted of paclitaxel and carboplatin combination and, during the relapse treatment, of gemcitabine. These regimens have been described in single cases to elicit a favorable response in the treatment of metastatic urachal carcinoma [5, 24, 27, 28]. However, no single chemotherapy regimen is to date proved effective enough to be considered a first-choice in all cases. Importantly, in the setting of salvage treatment, it is surgery that remains the most effective and potentially curative method [5]. Adjuvant chemotherapy in the cases of recurrent disease is usually applied. A multimodal treatment of metastatic urachal carcinoma has been shown to be capable of slowing disease progression and of providing over 10-year disease-free survival (in single cases) [7, 29, 30].

Our patient’s decision to submit to salvage surgery presented a major technical challenge. Since the diagnosis of liver metastasis until the surgery (a period of 2 months), the tumor grew from 4 to 8 cm and another metastatic focus in the liver was revealed. During hemihepatectomy, the tumor localization did not allow to obtain surgical margins considered sufficient (more than 1 cm), instead, resection within tumor pseudocapsule was performed. It is known that even such resection is potentially curative; however, in our case, the pathology report confirmed R1 type resection [31].

## Conclusions

Urachal carcinoma is an extremely rare and aggressive malignancy posing a significant diagnostic and therapeutic challenge. The surgical management of the disease remains the cornerstone of therapy. The present report is the first to describe a salvage surgery in the case of urachal carcinoma with liver metastasis. It emphasizes the need for a prompt diagnosis and surgical treatment of urachal carcinoma. As is the case with all urinary bladder tumors, hematuria as an alerting symptom is still too often ignored by both patients and primary care physicians. What is more, the described case should bring more attention to the need for performing pelvic lymph node dissection and the necessity for a close follow-up surveillance in cases of an advanced urachal carcinoma.

## Abbreviations

NOS: Not otherwise specified
PLND: Pelvic lymph node dissection
